# Bacterial Nanocellulose as a Scaffold for *In Vitro* Cell Migration Assay

**DOI:** 10.3390/nano11092322

**Published:** 2021-09-07

**Authors:** Milena Ugrin, Jelena Dinic, Sanja Jeremic, Sandra Dragicevic, Bojana Banovic Djeri, Aleksandra Nikolic

**Affiliations:** 1 Institute of Molecular Genetics and Genetic Engineering, University of Belgrade, Vojvode Stepe 444A, 11042 Belgrade, Serbia; milena.ugrin@imgge.bg.ac.rs (M.U.); sanjajeremic@imgge.bg.ac.rs (S.J.); sandra.d@imgge.bg.ac.rs (S.D.); bojanabanovic@imgge.bg.ac.rs (B.B.D.); 2 Department of Neurobiology, Institute for Biological Research “Sinisa Stankovic”—National Institute of Republic of Serbia, University of Belgrade, Bulevar Despota Stefana 142, 11060 Belgrade, Serbia; jelena.dinic@ibiss.bg.ac.rs

**Keywords:** bacterial nanocellulose, 3D cell culture, migration assay

## Abstract

Bacterial nanocellulose (BNC) stands out among polymers as a promising biomaterial due to its mechanical strength, hydrophilicity, biocompatibility, biodegradability, low toxicity and renewability. The use of scaffolds based on BNC for 3D cell culture has been previously demonstrated. The study exploited excellent properties of the BNC to develop an efficient and low-cost in vitro cell migration assay. The BNC scaffold was introduced into a cell culture 24 h after the SW480 cells were seeded, and cells were allowed to enter the scaffold within the next 24–48 h. The cells were stained with different fluorophores either before or after the introduction of the scaffold in the culture. Untreated cells were observed to enter the BNC scaffold in significant numbers, form clusters and retain a high viability after 48 h. To validate the assay’s usability for drug development, the treatments of SW480 cells were performed using aspirin, an agent known to reduce the migratory potential of this cell line in culture. This study demonstrates the application of BNC as a scaffold for cell migration testing as a low-cost alternative to commercial assays based on the Boyden chamber principle. The assay could be further developed for routine use in cancer research and anticancer drug development.

## 1. Introduction

Cell migration is involved in many biological processes such as embryonic development, the immune response, cancer metastasis and inflammation. Approaches to study cell migration are useful in the fields of physiology and oncology, particularly when studying the effect of novel therapeutic drugs [1]. The migration and invasion assays can provide the data needed for understanding the cell’s spontaneous migration, as well as its directional migration as a response to a chemoattractant [2]. One of the most widely used methods to study cellular migration and invasion is the transwell assay, which can be used to test various chemoattractants, such as chemokines, growth factors, lipids and nucleotides [3,4]. This approach can be further altered for investigating cell invasion by adding a layer of either an extracellular matrix or endothelial cells on top of the transwell membrane [5]. Another commonly used approach to study cell migration and invasion is microscopic live-cell imaging, but transwell assay chambers are more widely used due to their relatively low cost and moderate through-put capacity [6].

Bacterial nanocellulose (BNC) stands out among polymers as a promising biomaterial due to its mechanical strength, hydrophilicity, biocompatibility, biodegradability, low toxicity and renewability [7]. Dissimilar to nanocellulose obtained from plant sources, BNC does not require pretreatment to remove lignin and hemicellulose, as it is synthesized as pure cellulose with an ultra-fine network architecture, and characteristic ribbon-like microfibrils of 20–100 nm in diameter. This remarkable biopolymer has already found its purpose in the food, technology, electronic, papermaking and pharmaceutical industries, as well as biomedicine [8]. Out of all the mentioned applications, the one in the biomedical field attracts the most attention in both science and the industry. One of the best accepted clinical applications of BNC so far has been a topical wound dressing, while the potential of BNC application for medical implants, tissue engineering and drug delivery is great and still untapped [9,10,11,12]. Recently, BNC has emerged as a promising scaffold material for the adhesion and proliferation of cells and 3D bioprinting for tissue engineering purposes [13,14,15]. Biomedical applications of BNC rely largely on its network-like structure, which can be improved using chemical and genetic engineering tools [16,17,18,19].

BNC-based hydrogels have recently been explored as scaffolds for 3d cell culture that provide an environment for cell growth. The reproduction of in vivo conditions for cell growth in 3D requires a gelatinous and flexible matrix, and BNC has adequate characteristics to serve this purpose. The mechanical strength and flexibility of BNC scaffolds compensate for the most common disadvantages of peptide hydrogel matrices used in current 3D cell culture settings [20,21]. Additionally, the BNC scaffolds represent an easily produced low-cost alternative to expensive peptide hydrogel matrices that remain expensive due to difficulties in their production. After unsuccessful attempts to exploit decellularized tissues to isolate and reuse the extracellular matrix, and to develop a synthetic alternative to provide conditions similar to those found in vivo, nanocellulose hydrogels have gained attention with their ability to sustain 3D cultures of stem and cancer cells [22,23]. When grown in these scaffolds, cells show a high viability (over 90%) for several weeks in culture and no decrease in pluripotency.

Although it was thoroughly studied as a 3D cell culture scaffold, the potential of BNC for studying cellular migration remains unexplored. The study tested BNC as a scaffold for in vitro cell migration with a purpose of developing an efficient and low-cost assay that could be used in laboratory research and drug development.

## 2. Materials and Methods

### 2.1. Preparation of BNC Scaffold

BNC was produced and characterized as described previously using *Komagataeibacter medellinensis* ID13488 (CECT 8140 (Spanish Type Culture Collection)) [24,25]. The strain was cultivated in a standard Hestrin–Schramm medium pH 4.5 (HS) containing 20 g/L glucose, 5 g/L peptone, 5 g/L yeast extract, 2.5 g/L Na_2_HPO_4_ and 1.15 g/L citric acid, under static conditions for 8 days at 28 °C. BNC disks, obtained from growth in 24-well plates (well diameter 15.6 mm), were treated with 5% KOH aqueous solution and extensively washed with deionized H_2_O until a pH of 7.0 was reached. The estimated thickness of the wet scaffold was 700–900 µm. Before use in cell culture, BNC scaffolds were steam sterilized in autoclave at 121 °C for 15 min.

### 2.2. Cell Culture

The human colon adenocarcinoma SW480 cell line was maintained in a 5% CO_2_ humidified incubator at 37 °C. The cells were grown in Dulbecco’s Modified Eagle’s Medium (DMEM) (Capricorn Scientific, Ebsdorfergrund, Germany) supplemented with 10% fetal bovine serum (FBS) (Capricorn Scientific, Ebsdorfergrund, Germany) and 1% antibiotic/antimycotic solution (Capricorn Scientific, Ebsdorfergrund, Germany). Cells were subcultured using 1× trypsin/EDTA (Thermo Fisher Scientific, Waltham, MA, USA) after reaching 70–80% confluence.

For the migration assay, cells were trypsinized, washed twice with phosphate-buffered saline (PBS), seeded at a density of 500,000 cells in 6-well plates and left overnight to attach to the plate surface. The BNC scaffold was placed over the cells, fastened by adding the glass plate (4 cm^2^) and incubated with the cells for 24 h and 48 h. Cells were treated with aspirin as a known migration inhibition agent of cell line SW480 [26]. Concentrations of 1 mM and 2 mM were used, since they were shown to reduce migration of this cell line with little or no toxic effect. The treatment of cells with aspirin was performed just before the addition of the BNC scaffold. Migration of treated and untreated SW480 cells was evaluated by fluorescence and confocal microscopy.

### 2.3. Fluorescence Microscopy and Image Analysis

To observe cell morphology and localization, cells were stained with 10 µM CellTrace™ CFSE dye (Thermo Fisher Scientific, Waltham, MA, USA) for 30 min at 37 °C in the dark before seeding for the migration assay. After 24 h and 48 h, BNC scaffolds containing CellTrace™ CFSE-labeled SW480 cells were placed in a Nunc™ Glass Bottom Dish (Thermo Fisher Scientific, Waltham, MA, USA) in 1× PBS, and imaged at 5× magnification with a ZEISS Axio Vert inverted fluorescence microscope (Carl Zeiss Foundation, Jena, Germany) equipped with Axio Vision 4.8 software.

For quantification, SW480 cells in the BNC scaffold were labeled with 1 µg/mL Hoechst 33342 (Sigma-Aldrich Chemie Gmbh, Taufkirchen, Germany), a cell permeable nuclear stain, for 15 min at 37 °C after the migration assay. After washing in 1× PBS, the scaffold was placed in a Nunc™ Glass Bottom Dish in 1× PBS, and the cells were imaged at 5× magnification with a ZEISS Axio Vert inverted fluorescence microscope. Quantification of the cells in the scaffold was performed by counting the nuclei using the ImageJ package Fiji 1.53c (U.S. National Institutes of Health, Bethesda, MD, USA). Briefly, Hoechst 33342-labeled nuclei were marked using the threshold feature to distinguish the cells from the background. The Otsu method was used to set a threshold range. The Watershed algorithm was used to separate the touching particles. Next, the Analyze Particles function was used to count the number of particles (nuclei) in each image.

To evaluate cell viability, SW480 cells in the BNC scaffold were co-stained using Calcein AM (Tocris Bioscience, Bristol, UK) and Propidium iodide (Sigma-Aldrich Chemie Gmbh, Taufkirchen, Germany) as a LIVE/DEAD staining. The scaffolds containing cells were incubated for 15 min at 37 °C in medium with 4 μM Calcein AM, while Propidium iodide was added to a final concentration of 5 μM. After washing in 1× PBS, the scaffolds were placed in a Nunc™ Glass Bottom Dish in 1× PBS, and live (green) and dead (red) cells were imaged at 5× magnification with a ZEISS Axio Vert inverted fluorescence microscope. Live and dead cells in the scaffold were quantified using the ImageJ 1.53c software (U.S. National Institutes of Health, Bethesda, MD, USA). Briefly, the images were split into a single channel, 8-bit images. Then, the threshold was set to mark live (green channel) and dead (red channel) cells using the Otsu method, and the Watershed algorithm was used to separate the touching particles. Next, the Analyze Particles function was used to count the number of live and dead cells in each image.

### 2.4. Confocal Microscopy

The BNC scaffold containing CellTrace™ CFSE-labeled SW480 cells was placed in a Nunc™ Glass Bottom Dish in 1× PBS. Fluorescence microscopy images were taken using a Leica TCS SP5 II Basic confocal laser-scanning microscope (Leica Microsystems CMS GmbH, Mannheim, Germany) equipped with an Argon 488 nm laser and a 10× objective, using LAS AF 2.6 software (Leica Microsystems CMS GmbH, Mannheim, Germany). The emission of CellTrace™ CFSE dye was collected sequentially. The cells were imaged at every *z*-axis encompassing the BNC scaffold. All images were taken with a 512 × 512 pixel resolution and 8-bit color depth. Three-dimensional volume images were generated from the z-stack using the ClearVolume plugin in ImageJ software [27]. Three-dimensional volume images showed cells from the bottom to the maximum height reached in the BNC scaffold along the *z*-axis. Using the 3D viewer plugin in ImageJ software, a 360-degree rotation video of the 3D volume was created. The thickness of the z-stack used to create 360-degree rotation video was 285 μm.

### 2.5. Statistical Analysis

Statistical analysis was performed in GraphPad Prism 5.0 software (GraphPad Software, La Jolla, CA, USA). All groups were statistically compared to control by one-way analysis of variance (ANOVA) with Dunnett’s post hoc test. The accepted level of significance was *p* < 0.05.

### 2.6. Cost-Efficiency Analysis

The cost–efficiency model describes the average cost of the assay developed in this study against the cost of two products intended for the same purpose currently available on the market. The calculations were based on treating these three strategies as mutually exclusive, each associated with its costs and efficiency. Incremental cost for each product was calculated from market prices per sample analyzed. Incremental efficiency was defined as 2 for commercially available products used for migration and invasion analysis and as 1 for BNC scaffold as it can be used for migration analysis only. The incremental cost-efficiency ratio (ICER) was calculated as a ratio of incremental cost and incremental efficiency [28].

## 3. Results

### 3.1. Cellular Migration and Clustering in the BNC Scaffold

**Figure 1:**
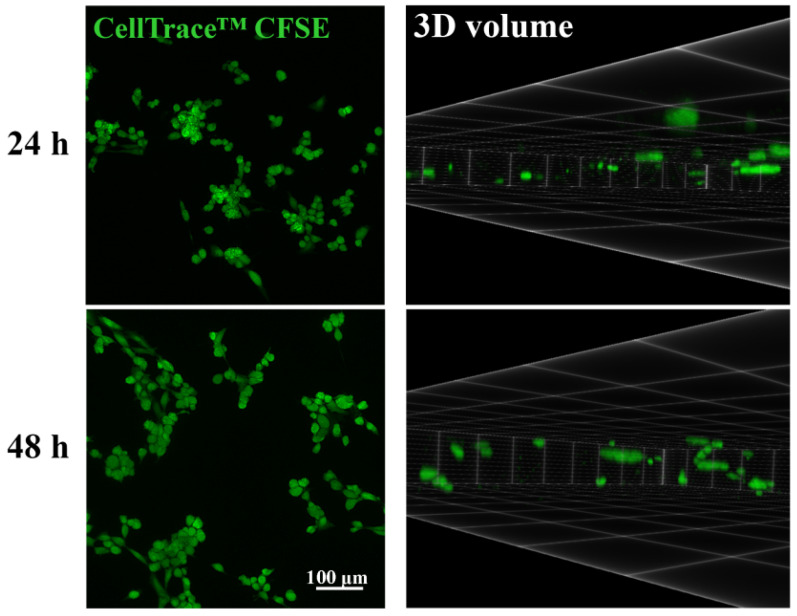
Morphology and localization of SW480 cells in the bacterial nanocellulose scaffold. Representative images (**left**) and 3D volume (**right**) of cells in the scaffold 24 h or 48 h after seeding. The thickness of the z-stacks used to create 3D volume images was 270 μm (24 h) and 285 μm (48 h). The cells were labeled with Cell Trace™ CFSE.

Untreated SW480 cells entered the BNC scaffold in significant numbers after 24 h, which was noticed after 48 h as well (Figure 1). It was also observed that cells were not distributed evenly and that they formed clusters upon entering the BNC scaffold (Figure 1, Supplementary Video S1, Supplementary Figure S1).

### 3.2. Viability of the Migrated Cells in the BNC Scaffold

**Figure 2:**
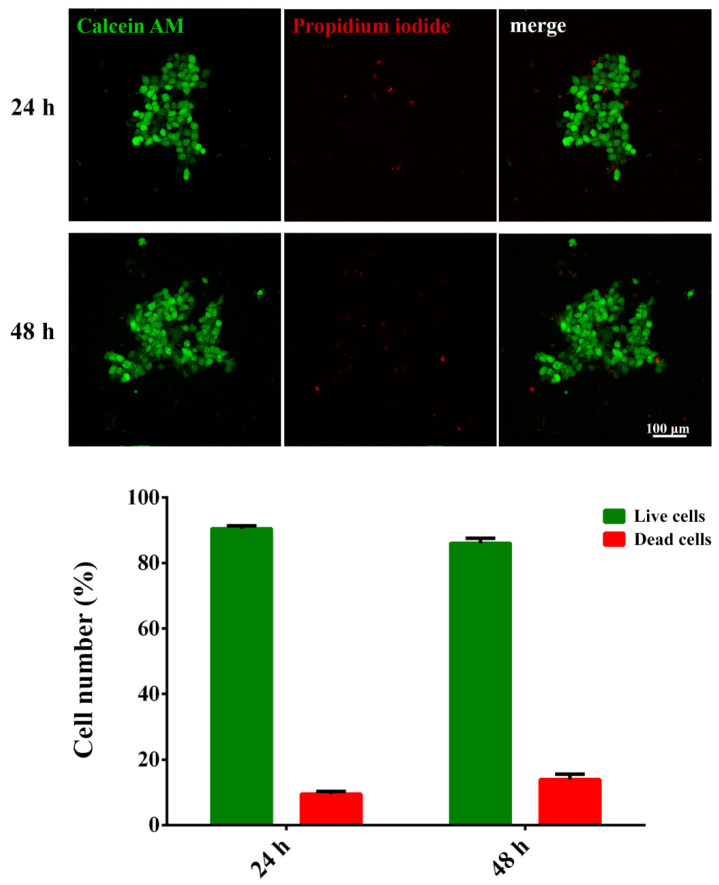
Viability of SW480 cells in the bacterial nanocellulose scaffold. Representative images and quantification of Calcein AM-labeled (**live**) and Propidium iodide-labeled (**dead**) cells in the scaffold 24 h or 48 h after the scaffold addition. The average number of live/dead cells per field was analyzed. Data are presented as mean ± SD.

The quantification of live and dead cells from the fluorescent micrographs showed that the viability of cells within the BNC scaffold was high, reaching around 90% after 24 h. The cells also retained high viability 48 h after adding the scaffold (Figure 2).

### 3.3. Cellular Migration in the BNC Scaffold under the Aspirin Treatment

**Figure 3:**
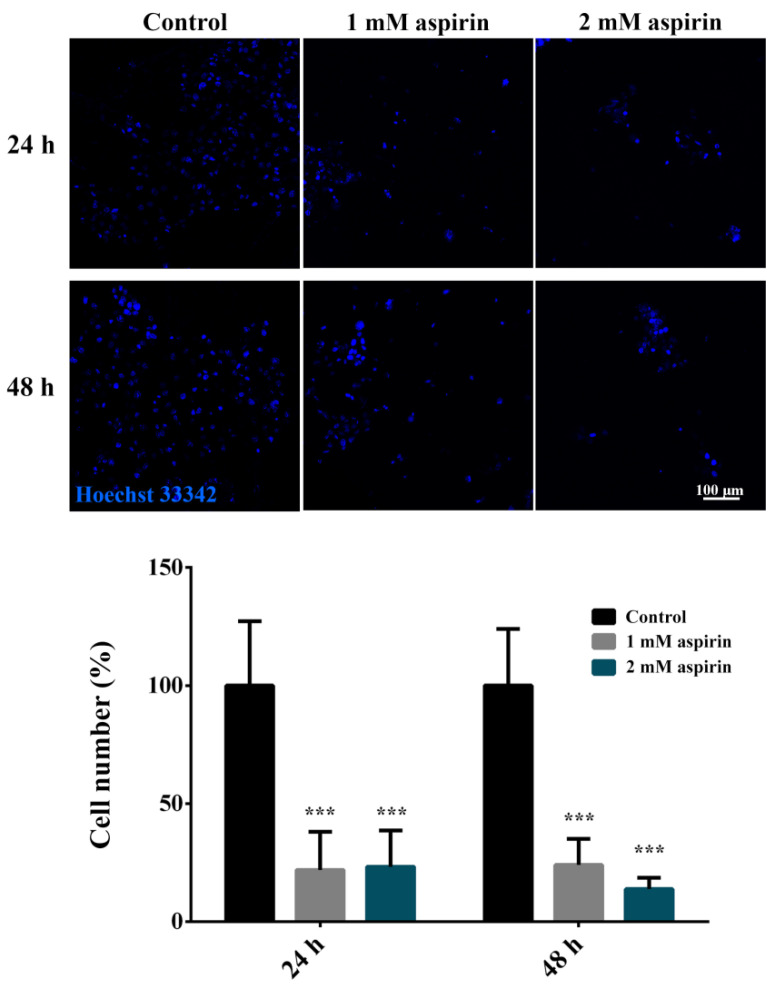
SW480 cell invasion into the bacterial nanocellulose scaffold after aspirin treatment. Representative images and quantification of cell migration into the scaffold 24 h or 48 h after treatment with aspirin. Cell nuclei were labeled with Hoechst 33342. The average number of nuclei per field was analyzed. Data are presented as mean ± SD. A statistically significant difference between treated and control groups is shown as *** (*p* < 0.001).

The treatments with 1 mM and 2 mM aspirin significantly decreased the number of cells that entered the BNC scaffold, reducing the number of migrated cells by approximately 80% (Figure 3).

### 3.4. Developed Assay for BNC-Based In Vitro Cell Migration Analysis

**Figure 4:**
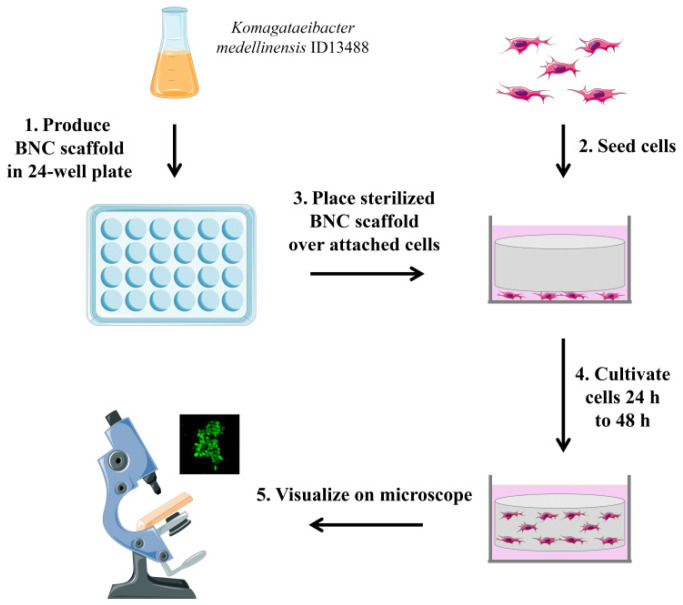
Schematic presentation of the developed in vitro cell migration assay based on bacterial nanocellulose. This figure was created with images adapted from Servier Medical ART (Servier, smart.servier.com (accessed on 20 June 2021), licensed under a Creative Commons Attribution 3.0 Unported License).

The overview of the developed assay is shown in Figure 4. The BNC scaffold disks were obtained by cultivating a BNC-producing strain in a standard medium in 24-well plates. Before use in a cell culture, BNC scaffolds were steam sterilized and transferred to tissue culture dishes containing attached cells. Cells were allowed to migrate into the BNC scaffold for 24–48 h. Cells in the BNC scaffold were then visualized and quantified using fluorescence microscopy. After incubation with an unused BNC scaffold, neither of the dyes stained the BNC and no background signal was detected. Therefore, different dyes can be used in the assay depending on the user and the experiment requirements.

### 3.5. Cost-Efficiency Analysis of the Developed Assay

The incremental cost was 0.25 EUR per sample for the BNC-based assay, since 5.92 EUR was the calculated price of the production of 24 scaffolds in a 24-well plate. The ICER value of the BNC-based assay was 10–52 times lower in comparison to the two analyzed commercially available assays.

## 4. Discussion

This study demonstrated the application of BNC as a scaffold for cell migration testing, which has not been previously reported in the literature. The described approach exploited excellent properties of BNC (mechanical strength, hydrophilicity, biocompatibility, low toxicity, renewability) to develop an efficient and low-cost assay suitable for routine use in life science laboratories.

The use of BNC enables the scaffold to be shaped into 3D structures by culturing bacteria in different molds, so the assay can be adjusted to various shapes and sizes of cell culture dishes to suit different types of experiments. In this study, BNC scaffolds were grown in 24-well plates and used for migration assay in 6-well plates, with a small glass plate used to press the scaffold to the bottom of the dish and, thus, secure the contact of the scaffold with the cell layer. The BNC used in this study was previously characterized showing ribbon-shaped nanofibrils ranging from 30 to 130 nm in width with a pore size estimated at 50 to 100 nm, with the typical morphology reported for BNC [25]. The assay was developed using SW480, a non-metastatic colon cancer cell line with a high migratory and invasive potential [29]. In addition to in vitro data, the high invasive potential of SW480 was confirmed by the fact that it was the precursor of the metastatic cell line SW620, derived from a lymph node metastasis of the same patient one year after the isolation of SW480 from his primary tumor [30]. To ensure that the cells detected within the scaffold were those that spontaneously migrated, the cells were allowed to adhere to the bottom of the flask before the migration test. In this study, BNC scaffolds were demonstrated to be non-cytotoxic and cells kept the expected high viability rate of above 85% after 48 h [31,32]. The height to which the cells migrated did not exceed 300 µm, which was approximately 30–40% of the maximum height, since the estimated thickness of the wet scaffold was 700–900 µm. The inhibition of cell migration was performed to confirm that the assay can be used for drug testing. To test the potential of BNC as a scaffold for a migration test, a well-characterized cell line, its known migration inhibitor and the previously established experimental conditions were used. Aspirin was applied in previously used concentrations of 1 mM and 2 mM, which were demonstrated not to cause cell death (in the case of the 1 mM concentration) or to cause a decrease in viability by 20% (in the case of the 2 mM concentration) [26].

Characteristics of BNC scaffolds overcame several problems imposed by the available assays. Of several different methods used to evaluate cell migration, the transwell Boyden chamber assay is most commonly used in laboratory practice [33]. Its main disadvantage is the relative difficulty in setting up the transwells. Commercially available cell culture inserts provide a solution to this problem, but they significantly increase the price of conducting such experiments. The membrane is usually coated with some extracellular matrix component, such as collagen, which facilitates cellular adherence and migration. Microfluidic migration devices overcome the limitations of the traditional transwell assay as they are designed to provide follow-up time of more than 48 h and enable real-time quantitative monitoring, but they additionally increase the cost of the experiment [34]. The scratch assay is another popular method for the study of cell migration, but scaling up this technique has proved challenging [35]. Dissimilar to the transwell Boyden chamber, BNC scaffolds were easily prepared and readily used for experiments. They also enabled long-term use with the option of real-time quantitative monitoring at a much lower cost than microfluidic devices. Additionally, due to the excellent properties of BNC, the scaffolds can be sterilized and reused, which could additionally decrease the cost of experiments in which the scaffolds are applied. Moreover, BNC is an environmentally friendly material, which could be easily decomposed after the intended end of usage. Due to its desirable characteristics, BNC has been explored not only as a scaffold for 3D cell cultures and tissue engineering, but also for other applications, such as the use as a carrier for drug delivery and a cryopreservable cell carrier for long-term storage and transport [36,37].

The proposed use of BNC scaffolds in their unaltered form was limited to testing of spontaneous cell migration. Cancer cells possess intrinsic mechanisms that drive their motility regardless of the environment and external stimuli, which make the proposed assay suitable for cancer research and drug development [38,39]. Our study demonstrated the usability of the developed assay as a low-cost strategy for the preliminary screening of a large number of samples when the effect of novel drugs and their combinations were tested. Its further development and eventual commercial use would require more comprehensive testing with different cell types. Its potential application for invasion testing would require the functionalization of BNC with the extracellular matrix components through chemical or genetic modifications [40,41]. However, the surface functionalization of BNC affects the cells by altering their attachment and survival, which may influence the obtained results when such scaffolds are used for invasion testing [18,42,43].

The possibility of cell staining with different fluorophores either before or after the introduction of the BNC scaffold in the culture allows the choice of different protocols for a qualitative and quantitative assessment of cell migration, depending on the requirements of the experiment. Staining of SW480 cells was performed using several different fluorescent dyes, and either of the applied dyes could be efficiently used for a BNC-based migration assay as neither stains the scaffold and no background signal was observed. The best and most practical approach entails the staining of the cells in the scaffold after the assay, just before imaging and dye Hoechst 33342 was successfully used for this purpose. Additional staining in this study was performed using Calcein AM and Propidium iodide for the assessment of cell viability. However, as the total number of migrated cells was of main interest for the assay results, live/dead staining is not necessary for routine purposes. If a follow-up for longer periods is of interest, the staining can be performed by Cell Trace™ CFSE that dyes live cells. Due to the covalent coupling reaction to intracellular molecules, CFSE can be retained within cells for extremely long periods and is not transferred to adjacent cells, which enables the fluorescence analysis at different time intervals [44].

The clustering of cells was observed in the BNC scaffold, which can be expected since cells grown in macroporous 3D scaffolds generally contain more cells growing in clusters [45]. It was demonstrated that the coating of BNC with different substances improved cell adhesion, growth and differentiation on the scaffold [46,47]. Although the cells adhered to and grew significantly more on composites, the uncoated BNC was an adequate choice for the migration assay, since it did not require the long-term cultivation of cells in the scaffold. Additionally, the lack of an active interface on the BNC scaffold appeared to allow cells to group in clusters; thus, reflecting a more natural behavior. This opens up possibilities for further development of BNC scaffolds toward use for the investigation of the cellular microenvironment.

The developed BNC-based assay for cell migration was an approach that yielded an excellent economic benefit, while securing the high level of efficiency equal to the available commercial products. The initial investment entails the procurement of the BNC-producer strain, which can be ordered from any bacterial culture collection. Since the production of BNC scaffolds was performed at laboratory-scale, there were no specific requirements in terms of equipment or other infrastructure. The preparation of scaffolds demands a relatively small amount of time and effort. The cost-efficiency analysis demonstrated that the relative cost of the BNC-based scaffold would be 0.25 EUR per sample, which significantly reduces the cost of analysis, considering that the price of currently available commercial assays is 2.5–13 EUR per sample. Considering the cost and effort of the BNC-based scaffold production, the developed assay harbors a considerable technology transfer potential.

## 5. Conclusions

The use of BNC for the analysis of cellular migration was superior to the commercial assays based on the Boyden chamber principle. The described assay lowered the cost significantly, while at the same time retained all the essential characteristics necessary for the follow-up of cellular migration and allowed a customizable approach for the intended research purposes. The developed concept opens the possibility for successful technology transfer toward routine use in laboratory research and drug development.

## Data Availability

The data presented in this study are available on request from the corresponding author.

## Supplementary Materials

The following is available online at https://www.mdpi.com/article/10.3390/nano11092322/s1, Video S1: Three-dimensional volume of Cell Trace™ CFSE-labeled SW480 cells in the bacterial nanocellulose scaffold 48 h after seeding, Figure S1: Representative 3D volume images of Cell Trace™ CFSE-labeled SW480 cells in the bacterial nanocellulose scaffold 48 h after seeding.
