# Supplementary material for: Bacterial Nanocellulose as a Scaffold for In Vitro Cell Migration Assay
Source: Nanomaterials (Basel). 2021 Sep 7;11(9):2322. doi: 10.3390/nano11092322 (PMC8468300; doi:10.3390/nano11092322)
Supplement: Supplementary file 1 [file nanomaterials-11-02322-s001.zip › nanomaterials-1311050-supplementary.pdf]

Supplementary Materials

Bacterial Nanocellulose as a Scaffold for *In Vitro* Cell Migration Assay

Milena Ugrin [1], Jelena Dinic [2], Sanja Jeremic [1], Sandra Dragicevic [1], Bojana Banovic Djeri [1] and Aleksandra Nikolic [1,*]

1 Institute of Molecular Genetics and Genetic Engineering, University of Belgrade, Vojvode Stepe 444A, 11042 Belgrade, Serbia; milena.ugrin@imgge.bg.ac.rs (M.U.); sanjajeremic@imgge.bg.ac.rs (S.J.); sandra.d@imgge.bg.ac.rs (S.D.); bojanabanovic@imgge.bg.ac.rs (B.B.D.)
2 Department of Neurobiology, Institute for Biological Research "Sinisa Stankovic"—National Institute of Republic of Serbia, University of Belgrade, Bulevar Despota Stefana 142, 11060 Belgrade, Serbia; jelena.dinic@ibiss.bg.ac.rs
* Correspondence: aleksni@imgge.bg.ac.rs

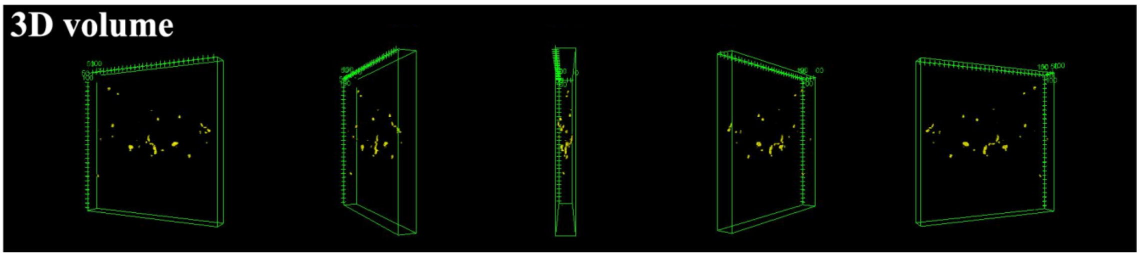

Figure S1. Representative 3D volume images of Cell Trace™ CFSE-labeled SW480 cells in the bacterial nanocellulose scaffold 48 h after seeding. The frames were taken from Supplementary Video S1. The thickness of the z-stack used to create 3D volume was 285 μm.
